# Microsatellite based genetic diversity of the widespread epiphytic lichen *Usnea subfloridana* (Parmeliaceae, Ascomycota) in Estonia: comparison of populations from the mainland and an island

**DOI:** 10.3897/mycokeys.58.36557

**Published:** 2019-08-30

**Authors:** Polina Degtjarenko, Inga Jüriado, Tiina Mandel, Tiiu Tõrra, Andres Saag, Christoph Scheidegger, Tiina Randlane

**Affiliations:** 1 Biodiversity and Conservation Biology, Swiss Federal Research Institute WSL, Zürcherstrasse 111, 8903, Birmensdorf, Switzerland Biodiversity and Conservation Biology, Swiss Federal Research Institute Birmensdorf Switzerland; 2 Department of Botany, University of Tartu, Lai 40, 51005, Tartu, Estonia University of Tartu Tartu Estonia

**Keywords:** Chemotypes, genetic diversity, environmental factors, lichenised fungi, microsatellites

## Abstract

Understanding the distribution of genetic patterns and structure is an essential target in population genetics and, thereby, important for conservation genetics. The main aim of our study was to investigate the population genetics of *Usnea subfloridana*, a widespread lichenised fungus, focusing on a comparison of genetic variation of its populations amongst three geographically remote and disconnected regions, in order to determine relationships amongst environmental data, variation in lichen secondary chemistry and microsatellite data in genotyped populations. In all, 928 *Usnea* thalli from 17 populations were genotyped using seven specific fungal microsatellite markers. Different measures of genetic diversity (allelic richness, private allelic richness, Nei’s unbiased genetic diversity and clonal diversity) were calculated and compared between lichen populations. Our results revealed a low genetic differentiation of *U. subfloridana* populations amongst three distant areas in Estonia and also a high level of gene flow. The results support suggestion of the long-range vegetative dispersal of subpendulous *U. subfloridana* via symbiotic propagules (soralia, isidia or fragments of thalli). Our study has also provided evidence that environmental variables, including mean annual temperature and geographical longitude, shape the genetic structure of *U. subfloridana* populations in Estonia. Additionally, a weak but statistically significant correlation between lichen chemotypes and microsatellite allele distribution was found in genotyped specimens.

## Introduction

The disentangling processes which shape genetic patterns and structure of natural populations is of great importance in understanding basic questions concerning evolution, ecology and conservation biology of species. The distribution of genetic diversity, which is a significant part of overall biodiversity, could indicate patterns of gene flow, genetic drift and potential for local adaptation (Frankham et al. 2010). The vast majority of previous studies about microsatellite diversity of lichenised fungi have used threatened, regionally rare or narrowly distributed lichens (e.g. Nadyeina et al. 2014; Jones et al. 2015; Prieto et al. 2015). However, the genetic diversity of common taxa could also be of particular interest since common species could be similarly susceptible to genetic consequences of habitat fragmentation as rare species (Honnay and Jacquemyn 2007). To date, only a few investigations have studied the genetic diversity of common and widespread lichenised fungi and genetic differentiation of their populations using microsatellite markers (e.g. Mansournia et al. 2012; Degtjarenko et al. 2018).

The epiphytic fruticose lichen *Usnea subfloridana* Stirt. has a wide distribution across Eurasia, Macaronesia and North America (Nash et al. 2007; Randlane et al. 2009; Smith et al. 2009). This is one of the commonest *Usnea* species in Estonia (Northern Europe) being frequently found on Norway spruce (*Picea abies*), Scots pine (*Pinus sylvestris*) and Silver birch (*Betula pendula*), as well as other deciduous trees and lignum (Tõrra and Randlane 2007; Randlane et al. 2011). Recent microsatellite studies of *U. subfloridana* populations indicated that unconstrained gene flow and exchange of multilocus genotypes existed between two geographically remote regions (the maximum distance between the two regions was 184 km) within the mainland of Estonia or had occurred at least in the past (Degtjarenko et al. 2018). Moreover, the natural habitat characteristics, such as stand age and mean circumference of the host tree, did not reveal any significant influence on measures of genetic diversity of *U. subfloridana* populations (Degtjarenko et al. 2016, 2018). However, some negative impact caused by alkaline dust pollution has been recorded on the genetic variation of this species (Degtjarenko et al. 2016).

Microsatellites or simple sequence repeats (SSR) are highly variable DNA sequences of short tandem repeats of 1–6 bp with co-dominant inheritance and appear as widely used markers for studying genetic variation and structure of natural populations (Goldstein and Schlötterer 1999; Ellegren 2004). The microsatellites are highly polymorphic and species-specific markers, considered as a most promising tool for investigating genetic diversity of highly clonal and complex organisms such as lichens (Werth 2010). The microsatellites were usually assumed to be neutral markers, occurring mainly in non-coding DNA (Ellegren 2004). Recent studies, however, have questioned this assumption, since microsatellites are also found in coding regions (e.g. Gemayel et al. 2012; Gao et al. 2013), playing a role in species adaptation and phenotypic plasticity within and across generations (Vieira et al. 2016).

Lichens produce a great number of extracellular secondary metabolites; these are synthesised by the mycobiont, although the carbon which is necessary for these substances is provided by the photobiont and subsequently transported to the fungus (Elix and Stocker-Wörgötter 2008). The production of polyketides, the most studied class of secondary metabolites in lichens, is regulated by polyketide synthases (PKS), the genes for which have been found in clusters (Shen 2003). Secondary metabolites of lichen-forming fungi are considered to have a distinct function, such as protecting the thalli against herbivores, pathogens or UV-radiation (Molnár and Farkas 2010). The presence or absence of specific secondary substances or their replacements by another substance has played an important role in identification and classification of these organisms when correlated with morphological or geographical differences (Elix and Stocker-Wörgötter 2008). Hence, variation of secondary compounds in lichens is probably not selectively neutral (Werth 2010). In *U. subfloridana*, three chemotypes have been reported (Halonen et al. 1998, 1999) while, in Estonia, two of them are known: (i) with thamnolic acid and (ii) with squamatic acid as the main substance in the medulla (Tõrra and Randlane 2007).

In the present research, we studied the population genetics of *U. subfloridana*, a widespread lichenised fungus, concentrating on a comparison of genetic variation of populations amongst three geographically remote and disconnected (by sea) regions. The main aims of our research were: (i) to study the genetic differentiation of *U. subfloridana* populations, growing in the south-eastern and northern regions of mainland and on a western island in Estonia, Northern Europe; (ii) to compare the measures of genetic diversity of *U. subfloridana* populations amongst the three study areas; (iii) to find whether allele frequencies in studied populations correlate with environmental variables; and (iv) to check if there were correlations between lichen chemotypes and microsatellite allele distribution in genotyped data.

## Material and methods

### Study area

**Figure 1.**
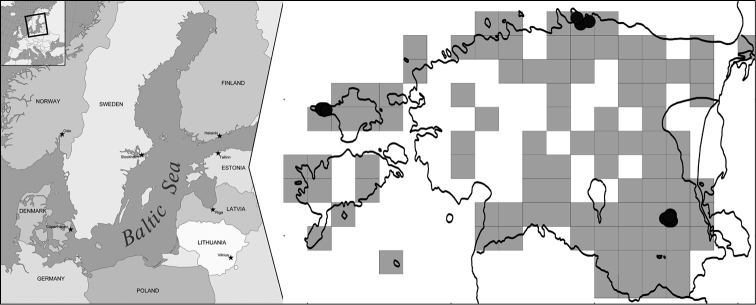
Distribution map of *Usnea subfloridana* in Estonia (light grey squares) and study populations (black circles) on Hiiumaa island in the western region (W), in the south-eastern region (SE) and in the northern region of Estonia; the map of Scandinavia was taken from free map resource http://d-maps.com/carte.php?num_car=5977&lang=en.

The study area is located in Northern Europe, in three geographically separate parts of Estonia: Lääne-Viru County, the northern region of mainland (hereafter N), Põlva County, the south-eastern region of mainland (hereafter SE) and Hiiumaa County, the second largest western island (hereafter W) of Estonia, located in the Baltic Sea (Fig. 1). According to climate norms from 1981 to 2010, N has a mean annual temperature of 5.7 °C, a mean annual precipitation of 587 mm, a mean wind speed of 3.9 m/s and a mean relative humidity of 80%, W has a mean annual temperature of 6.8 °C, a mean annual precipitation of 639 mm, a mean wind speed of 3.9 m/s and a mean relative humidity of 82% and SE has a mean annual temperature of 5.8 °C, a mean annual precipitation is 680 mm, a mean wind speed of 3.2 m/s and a mean relative humidity of 80% (Estonian Weather Service 2019). The vegetation of Estonia belongs to the hemiboreal forest zone, lying in the transitional area, where the southern taiga forest subzone changes into the spruce-hardwood subzone (Ahti et al. 1968; Laasimer and Masing 1995). The study sites from both geographical regions were situated in *Pinus sylvestris*-dominated boreal forests, being classified as the *Oxalis-Vaccinium myrtillus*, the *Vaccinium myrtillus*, the *Calamagrostio*-*Pinetum* and the *Vaccinium vitis-idaea* forest site types. These forest types are also widely distributed in other Baltic states (Kairiūkštis 1966; Bušs 1997), in Fennoscandia (Dierßen 1996) and in northwest Russia (Fedorchuk et al. 2005).

### Data collection

Fieldwork was carried out during the summer of 2011 (in SE), the autumn of 2014 (in N) and the autumn of 2016 (in W). The potential study sites for sampling were selected from forest survey maps using comparable forest characteristics (stand age and site type) from their forest survey (Forest Public Registry 2017). *Usnea subfloridana* populations, sampled from 17 study sites, three in N, eight in SE and six in W (Fig. 1; Table 1), were defined according to the boundaries of forest sites sharing the same values of forest survey data (forest site type, age of trees and proportion of trees in forest stand), according to the Forest Public Registry (2017). In each lichen population, an average of three *Usnea* thalli were collected from each Norway spruce tree, up to 6 m from the ground using a tree pruner. In total, 10–21 trees were surveyed and 30–66 thalli were randomly collected from each lichen population (Table 1). The tree circumference (BHC) was recorded for each sampled tree at breast height (1.3 m). The stand age was taken from the Forest Public Registry (2017).

**Table 1.** Characteristics of the studied *Usnea subfloridana* populations from the northern region (1–3), the southeastern region (4−11) and Hiiumaa island (12−17) of Estonia: sample size, geographical coordinates, tree variables, and measurements of genetic variation. Populations, the number of population; Specimens, the number of collected thalli per population; Trees, the number of host trees from which thalli were collected in each population; Latitude, latitudinal coordinates of the centre of forest site; Longitude, longitudinal coordinates of the centre of forest site; Age, the stand age (based on the oldest trees in the stands); BHC, mean circumference (cm) of the host tree per population (measured from each sampled tree at breast height 1.3 m); Squamatic acid, the number of collected thalli containing squamatic acid; Thamnolic acid, the number of collected thalli containing thamnolic acid; H, Nei’s unbiased genetic diversity per population; A, standardized allelic richness per population; G, the number of multilocus genotypes per population; M, clonal diversity per population; P, private allelic richness per population.

**Variables**	**Region**	**Northern (N)**			**Southeastern (SE)**					
	**Population**	**1**	**2**	**3**	**4**	**5**	**6**	**7**	**8**	**9**
**Sample size**	Specimens	46	30	36	52	60	60	58	60	57
	Trees	11	10	11	21	21	21	21	21	21
**Coordinates**	Latitude / Longitude	59°33'9.1"N, 25°48'4.1"E	59°35'54.6"N, 25°45'23.8"E	59°34'31.1"N, 25°55'47.7"E	58°6'13.5"N, 22°4'28.9"E	58°6'28.2"N, 22°2'50.4"E	58°7'13.2"N, 27°3'2.8"E	58°7'23.8"N, 26°59'6.0"E	58°7'23.0"N, 26°59'20.3"E	58°8'51.8"N, 27°3'16.2"E
**Tree variables**	Age	97	146	131	164	164	99	92	162	94
	BHC	93	119	90	125	117	136	77	119	84
**Chemotypes**	Squamatic acid	23	18	16	28	39	35	34	27	33
	Thamnolic acid	23	12	21	27	21	25	24	33	24
**Genetic variation**	H	0.58	0.60	0.63	0.65	0.62	0.65	0.62	0.62	0.63
	A	5.33	4.86	5.26	5.94	5.10	5.71	5.61	5.28	5.92
	G	38	27	31	42	50	46	45	50	45
	M	0.83	0.90	0.86	0.81	0.83	0.77	0.78	0.83	0.79
	P	0.13	0.04	0	0.28	0	0.11	0.03	0	0.05
**Variables**	**Region**	**Southeastern (SE)**		**Hiiumaa (W)**						**Total**
	**Population**	**10**	**11**	**12**	**13**	**14**	**15**	**16**	**17**	**17**
**Sample size**	Specimens	55	50	60	59	59	62	58	66	928
	Trees	20	20	20	20	21	21	21	21	322
**Coordinates**	Latitude / Longitude	58°8'29.3"N, 27°3'2.3"E	58°7'54.7"N, 27°2'28.3"E	58°55'45.7"N, 22°14'58.6"E	58°55'17.3"N, 22°14'39.3"E	58°55'55.5"N, 22°12'50.8"E	58°55'36.3"N, 22°12'07.4"E	58°55'52.2"N, 22°11'55.5"E	58°55'19.5"N, 22°15'10.6"E	
**Tree variables**	Age	94	174	96	167	167	157	96	96	
	BHC	119	107	74	130	110	135	86	80	
**Chemotypes**	Squamatic acid	26	22	27	36	31	28	29	25	477
	Thamnolic acid	29	28	33	23	28	34	29	41	455
**Genetic variation**	H	0.61	0.65	0.64	0.66	0.65	0.67	0.67	0.66	
	A	5.38	5.57	5.07	5.32	5.63	5.38	5.41	5.59	
	G	48	43	41	37	39	39	53	51	
	M	0.87	0.86	0.68	0.63	0.66	0.63	0.91	0.77	
	P	0.14	0	0.02	0	0.11	0.02	0	0.15	

### Chemical and molecular analyses

All collected *Usnea* thalli were air dried, cleaned to remove other lichen specimens and examined under a stereomicroscope. Thin layer chromatography (TLC) with solvent A (Orange et al. 2001) was used to confirm the identification of collected *Usnea* species. Then, 50 mg of each specimen was maintained in 1.5 ml microtubes at –20 °C until molecular analyses. The total genomic DNA was extracted using PowerPlant Pro DNA Isolation Kit and DNeasy Plant Mini Kit (MO BIO Laboratories, Inc., Qiagen, USA), according to the manufacturer’s protocol. Seven fungal microsatellite loci (*Us02*, *Us03*, *Us04*, *Us05*, *Us06*, *Us08* and *Us09*) were amplified in two multiplex PCR using QIAGEN Multiplex PCR Kit, following the instructions described in Tõrra et al. (2014) and Degtjarenko et al. (2016). Fragment lengths of PCR products were determined on a 3730xl DNA Analyzer (Applied Biosystems) with LIZ-500 as the internal size standard. The alleles were sized and genotyped using GeneMapper Software ver 5 (Applied Biosystems).

### Statistical analyses

The basic measurements of population genetics (the total number of alleles, mean number of alleles per locus, Nei’s unbiased genetic diversity (H) and allelic richness (A)) for *U. subfloridana* populations were calculated in the Microsatellite Analyzer ver 2.65 (MSA) (Dieringer and Schlötterer 2003). The measures of A were standardised using the rarefaction procedure implemented in the software MSA (Dieringer and Schlötterer 2003). The allelic richness of private alleles (P) per population was calculated using software HP-Rare (Kalinowski 2005). The number of multilocus genotypes (G), the percentage of multilocus genotypes, i.e. clonal diversity or genotypic diversity (M; the proportion of different genotypes in the population, G/N) and total number of multilocus genotypes from all populations were calculated in the software R (R Core Team 2013), using the R script by Werth et al. (2006). One-way analysis (ANOVA, type III) in the TIBCO Statistica ver 13.3 (TIBCO Software Inc.) was used to compare the different measurements of genetic diversity (A, H, P and M) amongst the three regions, N, SE and W.

The number of shared multilocus genotypes between populations was calculated in the software ARLEQUIN ver 3.5 (Excoffier and Lischer 2010). Clone correction of the genotyped dataset was performed in the software R (R Core Team 2013) using the R package ‘poppr’ (Kamvar et al. 2014; 2015). Hierarchical analyses of molecular variance (AMOVA) with 1023 permutations to estimate genetic differentiation were performed using ARLEQUIN ver 3.5 (Excoffier and Lischer 2010). The first, second and third AMOVA were performed at the tree level; genotyped individuals (364 multilocus genotypes and 124 trees) from populations of W, genotyped individuals (112 multilocus genotypes and 32 trees) from populations of N and genotypes individuals (452 multilocus genotypes and 166 trees) from populations of SE were analysed separately where each tree was treated as a distinct population. The fourth AMOVA was undertaken using all genotyped individuals (928 multilocus genotypes) and the fifth without identical multilocus genotypes or clone corrected dataset (403 multilocus genotypes). The rate of gene flow (Nm) across seven loci amongst 17 populations was estimated using GenAlex ver 6.5 (Peakall and Smouse 2012). Index of Association (Ia) was calculated to measure the extent of linkage equilibrium within a dataset by quantifying the amount of recombination amongst a set of sequences and observing association between alleles at different loci (Smith et al. 1993). The Ia was measured in the software R (R Core Team 2013) using the R package ‘poppr’ (Kamvar et al. 2014; 2015).

To assess the variation in data of *U. subfloridana* multilocus genotypes, the principal component analysis (PCA) was performed, implemented with the programme package Canoco 5.0 (Šmilauer and Lepš 2014). The data matrix of alleles in seven loci from 928 sample specimens was used. Variable ‘Lichen substance’ (squamatic or thamnolic acid) was used to group the samples. Subsequently, the redundancy analysis (RDA) (Šmilauer and Lepš 2014) was performed and nine explanatory variables were used to assess the correlation with accounted multilocus genotypes. The explanatory variables used in RDA were ‘Lichen substance’ and environmental variables as Latitude, Longitude, Stand Age, a mean BHC of sample trees per population, a mean annual temperature, a mean annual precipitation, a mean wind speed and a mean relative humidity for populations in the region.

To assess the significance of the associations between allele frequency in the populations and environmental variables, the second RDA was implemented in the same programme package. The data matrix of frequency of 62 alleles in 17 populations was used. The number of records of each allele in each population was counted and log-transformed for data analyses. The same environmental variables as in the first RDA were used as the explanatory variables. In both RDA models, the interactive forward selection procedure with randomisation tests was employed to select the most important environmental variables influencing variation in response data, retaining variables with an independent significant contribution at the p < 0.05 level. Subsequently, variation partitioning analysis (VPA) in the same programme was employed. The unique effects of statistically significant explanatory variables and the shared proportion of variation, explaining the distribution of multilocus genotypes (the first RDA) or allele frequency in populations (the second RDA), was calculated. The statistically significant contribution of variables was tested by the permutation test (Monte-Carlo permutation test, 4999 unrestricted permutations).

## Results

In total, 62 alleles at seven microsatellite loci, all polymorphic (Table 1), in 928 specimens from 17 *U. subfloridana* populations were recorded. The minimum number of alleles was four in locus *Us04* and the maximum was 15 in locus *Us03* and, on average, 2.8–9.1 were detected per locus across 17 populations. The mean number of alleles per population varied from 5.3 to 6.1 in populations on the western island in Estonia (W), from 5.3 to 6.2 in populations in the south-eastern region of mainland (SE) and from 4.9 to 5.4 in populations in the northern region of the mainland (N). There were 403 different multilocus genotypes across 928 genotyped specimens in 17 lichen populations. Allelic richness (A) varied from 4.86 to 5.94 across all lichen populations and Nei’s unbiased genetic diversity (H) ranged from 0.58 to 0.67 (Table 1). Other detailed measurements of genetic diversity per population are given in Table 1. The mean gene flow (Nm) for all populations across seven loci was 7.29.

The results of ANOVA showed that Nei’s unbiased genetic diversity (H) depended significantly on the region (*F* (2, 12) = 10.74, *p* = 0.001); H was higher in populations from W and lower in populations from N. The clonal diversity (M) also depended significantly on the region (*F* (2, 14) = 5.62, *p* = 0.02); M was higher in populations from N and lower in populations from W. The allelic richness (A; *F* (2, 14) = 2.83, *p* = 0.09) and private allelic richness (P; *F* (2, 14) = 0.18, *p* = 0.83 did not differ amongst the three regions (N, SE and W).

**Figure 2.**
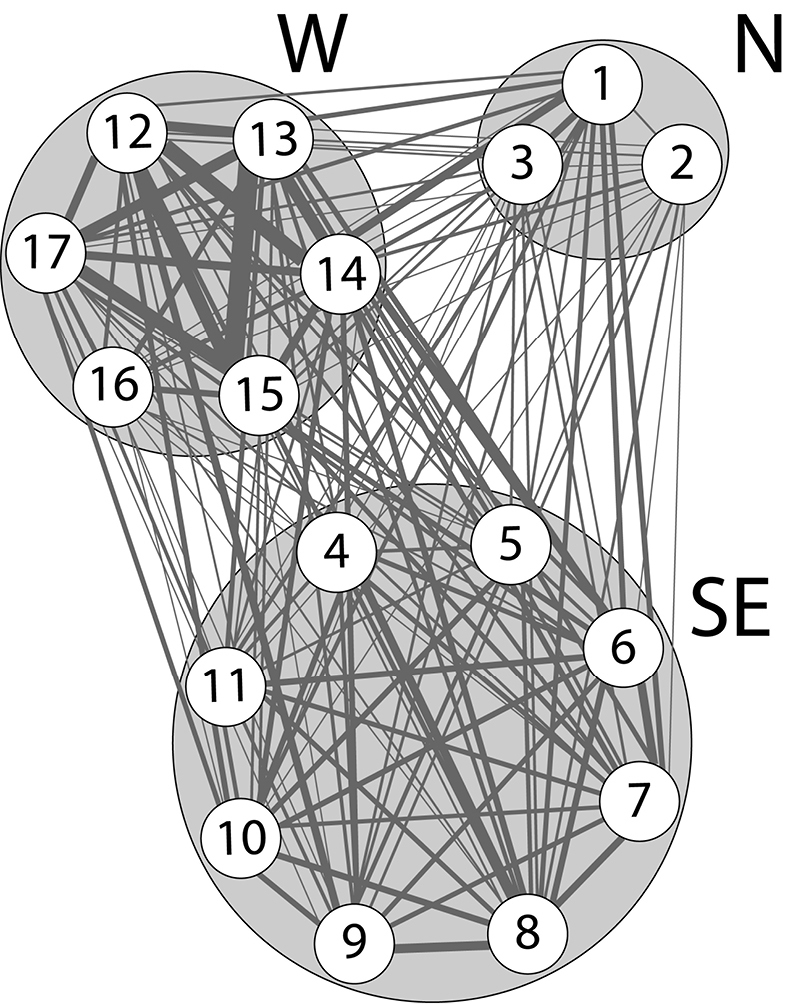
Total number of shared haplotypes between populations of *Usnea subﬂoridana* in the south-eastern (SE), the western (W) and northern (N) regions of Estonia; the thickness of lines reﬂects the number of shared haplotypes between populations.

The analyses for checking shared haplotypes amongst populations in the software ARLEQUIN ver 3.5 indicated that all *Usnea* populations shared the identical multilocus genotypes with other populations, as well as amongst three regions, N, W and SE (Fig. 2; Suppl. material 1). The Index of Association (Ia) differs significantly from zero which means that *Usnea* multilocus genotypes are likely undergoing clonal reproduction in study populations (Kamvar et al. 2014; 2015). The first, second and third AMOVA results showed that most of the total genetic variation (96.1% in W, 97.5% in SE and 95.1% in N) was due to the differences amongst individuals within study tree and 0.5%, 0.3% and 0.7% of genetic variation (for W, SE and N, respectively) was found amongst populations (Table 2). The fourth AMOVA results (928 genotypes) revealed that most of the total genetic variation (97.7%) was due to the differences amongst individuals within studied *Usnea* populations; a low proportion (1.8%) of genetic variation was attributed to regional differences (Table 2). The results from the fifth AMOVA (clone corrected, 403 genotypes) showed that most of the total genetic variation (98.7%) was also due to differences amongst individuals within studied *Usnea* populations and 1.2% of genetic variation was found between the regions (Table 2).

**Table 2.** Results of hierarchical analyses of molecular variance (AMOVA) for 17 populations of *Usnea subfloridana* according to seven microsatellite loci with 364 multilocus genotypes from populations on the western island in Estonia (W), with 452 multilocus genotypes from populations in the south-eastern region of the mainland (SE), with 112 multilocus genotypes from populations in the northern region of the mainland (N), with all multilocus genotypes (928 specimens) and clone corrected dataset (403 specimens). Values of P, in bold, represent a significant effect; d.f., the number of degrees of freedom.

**Source of variation**	**d.f.**	**Sum of squares**	**Variance**	**Percentage** %	**P**
I AMOVA (364 multilocus genotypes and 124 trees)					
Amongst regions (i.e. populations)	5	15.9	0.01	0.5%	**0.005**
Amongst populations within regions (i.e. amongst trees)	118	292.8	0.08	3.4%	**0.008**
Within populations (i.e. trees)	240	539.0	2.25	96.1%	**0.08**
Total	363	847.7	2.3		
II AMOVA (452 multilocus genotypes and 166 trees)					
Amongst regions (i.e. populations)	7	18.8	0.007	0.3%	**0.02**
Amongst populations within regions (i.e. amongst trees)	158	365.5	0.05	2.3%	**0.04**
Within populations (i.e. trees)	286	622.5	2.2	97.5%	0.1
Total	451	1006.9	2.2		
III AMOVA (112 multilocus genotypes and 32 trees)					
Amongst regions	2	6.04	0.02	0.7%	**0.02**
Amongst populations within regions	29	68.3	0.09	4.2%	**0.04**
Within populations	80	163.9	2.05	95.1%	0.13
Total	111	238.2	2.2		
IV AMOVA (928 genotypes)					
Amongst regions	2	28.1	0.04	1.8%	**<0.001**
Amongst populations within regions	14	40.7	0.01	0.5%	**0.001**
Within populations	911	2052.6	2.5	97.7%	**<0.001**
Total	927	2121.5	2.3		
V AMOVA (403 genotypes)					
Amongst regions	2	11.5	0.03	1.2%	**0.007**
Amongst populations within regions	14	33.6	0.002	0.03%	0.389
Within populations	386	906.6	2.3	98.7%	**<0.001**
Total	402	951.7	2.4		

**Figure 3.**
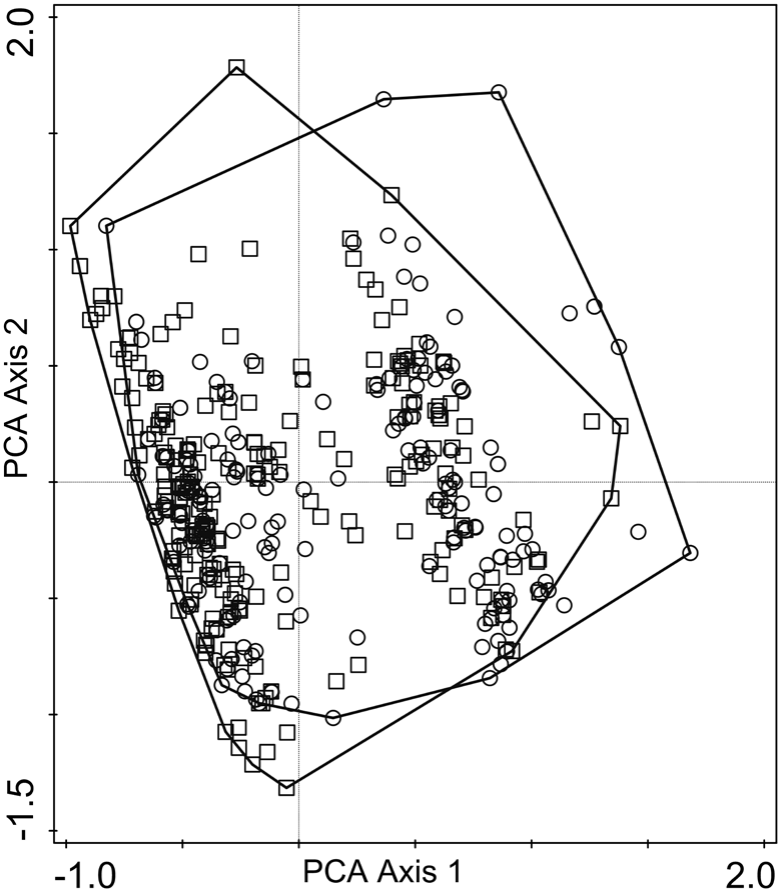
*Usnea subfloridana* multilocus genotypes in the principal component analysis (PCA) ordination plot of the first and second axes. Samples are grouped according to the presence of lichen substance: samples containing thamnolic (square) or squamatic acid (circle).

**Figure 4.**
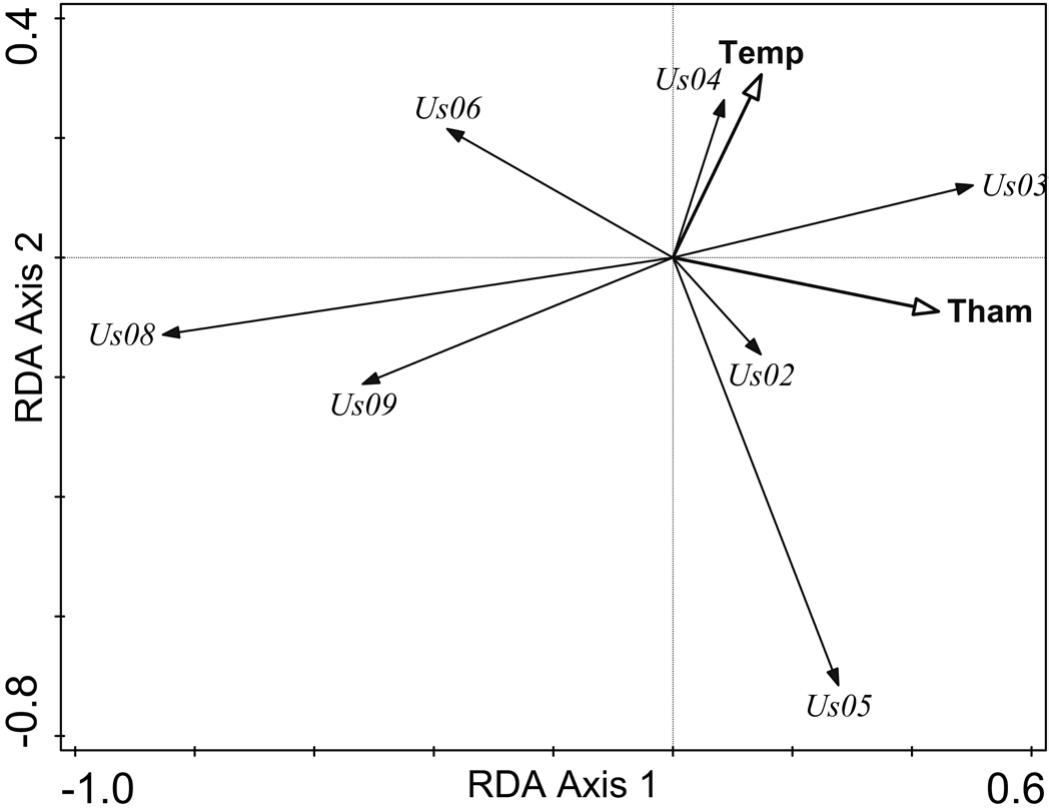
*Usnea subfloridana* multilocus genotypes (Us02, Us03, Us04, Us05, Us06, Us08, Us09) and explanatory variables mean annual air temperature (‘Temp’) and the presence of thamnolic acid (‘Tham’) in a lichen sample in the bi-plot of the redundancy analysis (RDA) of the first and second axes.

In the PCA ordination of multilocus genotypes of *U. subfloridana*, the first ordination axis accounted for 34.5% and the second axis for 20.5% of variation in the sample data. The sampled specimens constituted a rather homogenous cluster in the PCA ordination plot and only a minor distinction, according to the presence of thamnolic or squamatic acid, was visible (Fig. 3). In the RDA analyses with nine explanatory variables, the model accounted for 5.7% of variation in the response data. According to the results of the interactive forward selection of explanatory variables, the variables ‘Lichen substance’ and ‘Temperature’ contributed significantly to the explanation of variation in the response data (Fig. 4). The other environmental variables did not make a significant contribution to the model and were left out during the interactive forward selection of variables (*p* ≥ 0.05). The results of variation partitioning analysis (VPA) showed that two variables, ‘Lichen substance’ and ‘Temperature’, represent 5.5% of the variation of *U. subfloridana* multilocus genotypes (adjusted variation), the former being 4.1% and the latter 1.3% of the total variation, while the co-effect of both variables was less than 0.1% (*p* = 0.0005).

**Figure 5.**
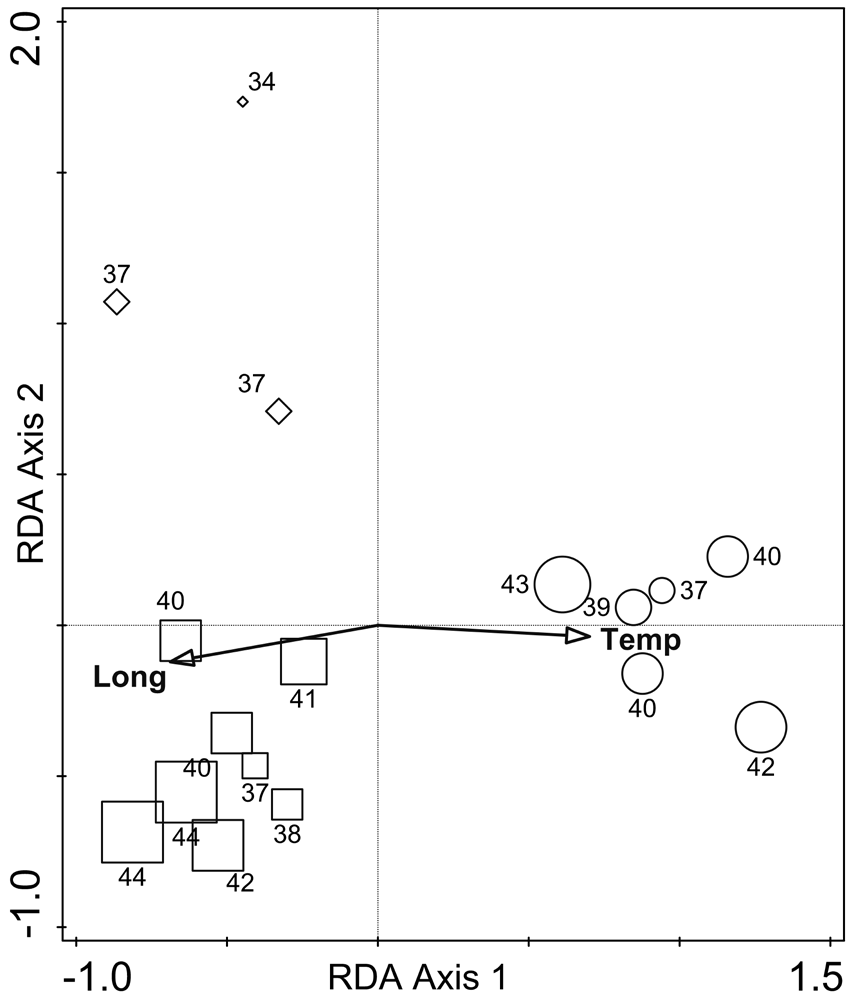
Sample populations of *Usnea subfloridana* and explanatory variables mean annual air temperature (‘Temp’) and geographical longitude of populations (‘Long’) in the bi-plot of the redundancy analysis (RDA) of the first and second axes. The shape of symbols indicates the geographical location of studied populations (square – south-eastern region of mainland, circle - western island and diamond – north-eastern region) and the size of symbols indicates the number of different alleles found in the studied populations.

**Figure 6.**
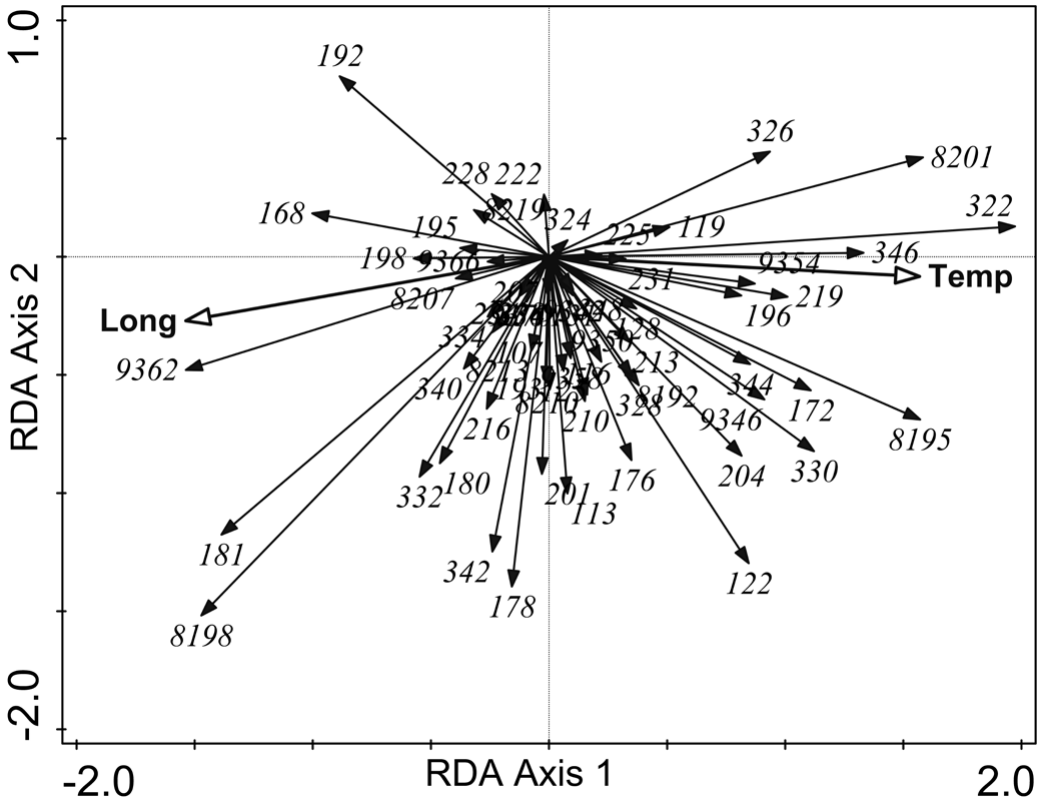
Alleles of *Usnea subfloridana* and explanatory variables mean annual air temperature (‘Temp’) and geographical longitude of populations (‘Long’) in the bi-plot of the redundancy analysis (RDA) of the first and second axes. Labels of alleles prefixed by ‘8’ or ‘9’ indicate that these alleles belong to loci *Us08* or *Us09*, respectively; for example, 8201 means that allele 201 is from *Us08*

The model of the second RDA with allele frequency data in studied populations accounted for 36.1% of variation in the response data. According to the results of the interactive forward selection of explanatory variables, the variables ‘Temperature’ and ‘Longitude’ contributed significantly to the explanation of variation in the response data (Fig. 5), the other explanatory variables not making a significant contribution to the model and were left out during the interactive forward selection of variables (*p* ≥ 0.05). The results of variation partitioning analysis (VPA) showed that the two explanatory variables, ‘Temperature’ and ‘Longitude’, represent 27.0% of the variation of allele frequency data (adjusted variation), the former being 8.2% and the latter 7.6% of the total variation, while the co-effect of both variables was 11.2% (*p* = 0.0005). Alleles, occurring only (e.g. 326 and 346 (*Us05*), 201(*Us08*, coded as 8201)) or being more frequent (e.g. 322 and 330 (*Us05*), 195(*Us08*), 345 (*Us09*)) in populations of western Estonia with an average higher air temperature, are located in the positive side of the ordination plot. The alleles, occurring only (e.g. 362 (*Us09*)) or being more frequent (e.g. 168 and 180 (*Us02*), 181 (*Us04*), 332 (*Us05*), 198 (*Us08*)) in populations of south-eastern Estonia, are located on the negative side of the ordination plot (Fig. 6).

## Discussion

Lichen-forming fungi, reproducing purely sexually, are assumed to have a longer dispersal distance and exhibit less genetic structure than clonally reproducing species via isidia/soredia or fragments of thalli (Werth 2010; Singh et al. 2015; Alors et al. 2017). However, our study showed that *Usnea subfloridana*, which usually reproduces asexually by symbiotic propagules, exhibited a very long dispersal range and a negligible level of genetic differentiation in populations from three geographically remote areas, namely the south-eastern (SE) and northern (N) regions of the Estonian mainland and Hiiumaa (W), an island in the Baltic Sea (Fig. 1; Table 2). Small-scale AMOVA also exhibited similar results; a negligible level of genetic differentiation was found amongst populations in three study regions (Table 2). Using highly variable microsatellite markers, our study also showed high levels of gene flow (Nm = 7.29) or genetic similarity amongst all studied *U. subfloridana* populations. Moreover, our results demonstrated that *Usnea* population shared common identical multilocus genotypes amongst all studied populations and also amongst the three regions (Fig. 2; Suppl. material 1). Our previous study also recorded a very low genetic differentiation of *U. subfloridana* populations between two distant areas in Estonia, suggesting spatially unrestricted dispersal of individuals and unconstrained gene flow in *U. subfloridana* populations (Degtjarenko et al. 2018). However, the genetic variation, attributed to regional differences, was slightly higher in the current study (1.8% and 1.2%; Table 2) than in the previous microsatellite study (0.5%) concerning *U. subfloridana* populations in Estonia (Degtjarenko et al. 2018). A possible explanation for this is that the maximum distance amongst the populations was longer (viz. the maximum distance was 295 km in the current and 184 km in the previous study) and, in addition, one region (W) with six populations was relatively isolated, i.e. an island. Similar levels of genetic differentiation at local scales have been indicated in previous studies; for example, for populations of *Lobaria pulmonaria* L., a lichen-forming fungus that also reproduces predominantly asexually (e.g. Walser 2004; Scheidegger et al. 2012). According to the test of Index of Association (Ia), our study found that multilocus genotypes of *U. subfloridana* in our localities most likely originated from asexual reproduction (Suppl. material 2). Therefore, it appears that the long-range dispersal of subpendulous *U. subfloridana* occurred via symbiotic propagules (soralia, isidia or fragments of thalli) that had to travel several kilometres over the sea.

The genetic diversity of natural populations is shaped by cumulative synergy of historical and present-day processes (Hewitt 2000; Frankham et al. 2010). Previous studies have shown that the climatic and habitat heterogeneity could be important in explaining the levels of genetic diversity of lichen populations; for example, annual precipitation had an effect on genetic diversity of *Lobaria pulmonaria* populations in the Iberian Peninsula (Otálora et al. 2015). Nadyeina et al. (2014) demonstrated that the microclimatic factors (air humidity and temperature) influenced the distribution of gene pools of *L. pulmonaria* in the Carpathian Mountains. The genetic diversity of *L. pindarensis* Räsanen was significantly influenced by altitude, revealing higher levels of genetic diversity at a high elevation in the Himalayas (Devkota et al. submitted). Belinchón et al. (2018) showed that *Nephroma parile* (Ach.) Ach. and *N. laevigatum* Ach. were also related to measured environmental and habitat variables, indicating micro-evolutionary responses to the environment. Our studies showed that genetic diversity (H) of *Usnea subfloridana* populations was higher on Hiiumaa island while clonal diversity (M) was higher in the northern region (N) of Estonia. This is contrary to our *a priori* assumption that relatively isolated populations, occurring on islands, will exhibit a lower genetic diversity and higher levels of clonal diversity. Moreover, the allele distribution in studied populations correlated with a mean annual temperature and geographical longitude (Fig. 5). These findings suggest (in our study – average annual temperature and longitude) are important factors for shaping the genetic structure and patterns of *U. subfloridana* populations in Estonia and indicating local adaptation to landscape conditions. The results of the redundancy analysis (RDA) indicated that, for example, several alleles occurring only or being more frequent are found in the populations of western Estonia with a higher average air temperature while other alleles, occurring only or being more frequent in populations of south-eastern Estonia, are related to a lower average air temperature (Fig. 6). Habitat quality measured as age of stand or host tree could be important in explaining the distribution of genetic diversity of lichen populations (Jüriado et al. 2011; Otálora et al. 2011; Scheidegger et al. 2012). However, RDA showed that the age of forest stands and circumference of host trees did not have a significant effect on the distribution of allele frequencies in the studied populations. This also accords with our earlier observations, which showed that stand age or tree circumference were not of great importance in explaining the genetic patterns of *U. subfloridana* populations (Degtjarenko et al. 2016, 2018). Overall, our results reveal that, despite extensive gene flow and low genetic differentiation amongst the distant areas, geographical longitude and temperature heterogeneity might promote the current levels of genetic diversity of *U. subfloridana* populations amongst these three remote regions in Estonia.

Recent studies highlighted that microsatellites could be found in coding regions and be linked with adaptation and phenotypic consequences (e.g. Gemayel et al. 2012; Gao et al. 2013). In our study, for the first time any probable correlations between the chemotypes of a lichen-forming fungus, *U. subfloridana* and its microsatellite allele distribution in genotyped data were checked. Both chemotypes of *U. subfloridana* are common in Estonia (Tõrra and Randlane 2007) and nearly evenly represented in study regions (Table 1). The results of this study showed that only a minute difference, according to the presence of thamnolic or squamatic acid, was visible (Fig. 3) in sampled specimens, representing a rather homogenous cluster in the PCA ordination plot (Fig. 3). Recently, Lagostina et al. (2018) showed that chemotypes were not correlated with any of the genetic clusters of the two morphotypes, *U. antarctica* Du Rietz and *U. aurantiacoatra* (Jacq.) Bory, in the study delimiting species within the genus Usnea subgenus Neuropogon. However, the chemical variation across these species was not randomly distributed between the two morphotypes nor amongst the genetic clusters (Lagostina et al. 2018). The presence of particular secondary compounds is also known to be specific to a certain habitat or ecoregion and to respond to the environmental gradients (e.g. Nash and Zavada 1977; Culberson et al. 1977; Zhou et al. 2006). We speculate that the correlation between particular microsatellite alleles and secondary metabolite could be relevant to the adaptation of populations to a certain geographical region and contribute thus to the minute isolation by geographical distance.

## Conclusion

We studied the population genetics of *U. subfloridana*, a widespread lichenised fungus, concentrating on a comparison of genetic variation of populations amongst three geographically remote and disconnected (by sea) regions in Estonia. We recorded a very low genetic differentiation of *U. subfloridana* populations amongst three distant areas, suggesting spatially unrestricted dispersal of individuals and unconstrained gene flow in *U. subfloridana* populations. Furthermore, geographical longitude and the mean annual temperature might play an important role in forming genetic variation in *U. subfloridana* populations in Estonia. This work contributes to the existing knowledge of population genetics of highly clonal and complex organisms, such as lichens.

## Supplementary materials

Supplementary material 1 Link: https://doi.org/10.3897/mycokeys.58.36557.suppl1 https://binary.pensoft.net/file/329668This dataset is made available under the Open Database License (http://opendatacommons.org/licenses/odbl/1.0/). The Open Database License (ODbL) is a license agreement intended to allow users to freely share, modify, and use this Dataset while maintaining this same freedom for others, provided that the original source and author(s) are credited.Polina Degtjarenko, Inga Jüriado, Tiina Mandel, Tiiu Tõrra, Andres Saag, Christoph Scheidegger, Tiina Randlane

Supplementary material 2 Link: https://doi.org/10.3897/mycokeys.58.36557.suppl2 https://binary.pensoft.net/file/329669This dataset is made available under the Open Database License (http://opendatacommons.org/licenses/odbl/1.0/). The Open Database License (ODbL) is a license agreement intended to allow users to freely share, modify, and use this Dataset while maintaining this same freedom for others, provided that the original source and author(s) are credited.Polina Degtjarenko, Inga Jüriado, Tiina Mandel, Tiiu Tõrra, Andres Saag, Christoph Scheidegger, Tiina Randlane
