# Supplementary figures and images for: Microsatellite based genetic diversity of the widespread epiphytic lichen Usnea subfloridana (Parmeliaceae, Ascomycota) in Estonia: comparison of populations from the mainland and an island
Source: MycoKeys. 2019 Aug 30;58:27–45. doi: 10.3897/mycokeys.58.36557 (PMC6731264; doi:10.3897/mycokeys.58.36557)

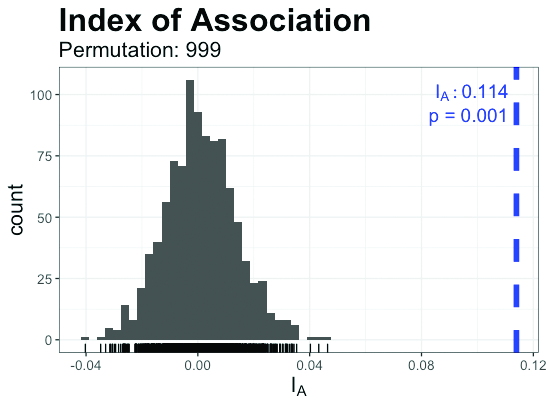

Supplement: Supplementary material 1 [file mycokeys-58-027-s002.tif]
